# Loss of GATA6-mediated up-regulation of UTX promotes pancreatic tumorigenesis and progression

**DOI:** 10.1016/j.gendis.2023.01.019

**Published:** 2023-03-31

**Authors:** Hui-Qing Zhang, Fanyang Kong, Xiangyu Kong, Tingting Jiang, Muyuan Ma, Shaojiang Zheng, Junli Guo, Keping Xie

**Affiliations:** a The Third Department of Medical Oncology, Jiangxi Cancer Hospital, Nanchang, Jiangxi 330006, China; b Departments of Gastroenterology, Changhai Hospital, Second Military Medical University, Shanghai 200433, China; c Center for Pancreatic Cancer Research, The South China University of Technology School of Medicine, Guangzhou, Guangdong 510006, China; d Hainan Clinical Medical Research Center of the First Affiliated Hospital, Hainan Women and Children's Medical Center, Hainan Medical University, Haikou, Hainan 570102, China; e Key Laboratory of Tropical Cardiovascular Diseases Research of Hainan Province, Key Laboratory of Emergency and Trauma of Ministry of Education, Hainan Medical University, Haikou, Hainan 571199, China

**Keywords:** Growth, Invasion, Metastasis, Pancreatic cancer, UTX

## Abstract

Ubiquitously transcribed tetratricopeptide repeat on chromosome X (UTX), also known as lysine (K)-specific demethylase 6A (KDM6A), functions as a tumor suppressor gene or oncogene depending on the tumor type and context. However, its tumor-suppressive mechanisms remain largely unknown. Here, we investigated the clinical significance and biological effects of UTX expression in pancreatic ductal adenocarcinoma (PDA) and determined the potential mechanisms of its dysregulation. UTX expression and its association with clinicopathologic characteristics of PDA patients were analyzed using immunohistochemistry. UTX mRNA and protein expression and their regulation in PDA cell lines were measured using quantitative polymerase chain reaction and Western blot analyses. The biological functions of UTX in PDA cell growth, migration, and invasion were determined using gain- and loss-of-function assays with both *in vitro* and *in vivo* animal models. UTX expression was reduced in human PDA cell lines and specimens. Low UTX expression was associated with poor differentiation and prognosis in PDA. Forced UTX expression inhibited PDA proliferation, migration, and invasion *in vitro* and PDA growth and metastasis *in vivo*, whereas knockdown of UTX expression did the opposite. Mechanistically, UTX expression was trans-activated by GATA6 activation. GATA6-mediated PDA progression could be blocked, at least partially, by silencing UTX expression. In conclusion, loss of GATA6-mediated UTX expression was evident in human PDA and restored UTX expression suppressed PDA growth and metastasis. Thus, UTX is a tumor suppressor in PDA and may serve as a prognostic biomarker and therapeutic target.

## Introduction

Pancreatic ductal adenocarcinoma (PDA), generally known as pancreatic cancer, is the seventh leading cause of cancer-related deaths worldwide.^1^, ^2^, ^3^ In the United States, the incidence of PDA is increasing, with an estimated 53,070 new cases and 41,780 PDA-related deaths in 2016.^2^, ^3^, ^4^, ^5^ Recently, surgical resection, radiation therapy, chemotherapy, and immunotherapy for PDA have improved, although its 5-year survival rate remains less than 8%.^3^, ^4^, ^5^, ^6^, ^7^, ^8^ Thus, further studies of the molecular mechanisms of PDA development and progression and identification of new therapeutic targets for PDA are urgently needed.^9^, ^10^, ^11^, ^12^, ^13^

Our knowledge of PDA development and progression has advanced significantly because of a rapid increase in our understanding of PDA molecular biology and genetics.^14^, ^15^, ^16^, ^17^, ^18^ Existing data provide global insights into genetic alterations in and molecular profiles of PDA.^15^, ^16^, ^17^, ^18^, ^19^, ^20^ Established inherent and acquired genetic and epigenetic alterations in PDA include mutations of the oncogene *K-ras* and inactivation of the tumor suppressors *TP53* and *DPC4*.^15^, ^16^, ^17^, ^18^, ^19^ Consequently, various signaling pathways are deregulated in PDA cells (*e.g.*, Wnt/β-catenin, Krüppel-like factor 4, and Forkhead box M1 signaling axes).^18^, ^19^, ^20^, ^21^, ^22^ Presumably, these alterations critically promote cellular transformation and tumor initiation and progression.^21^, ^22^, ^23^ However, potential new driver pathways for PDA progression remain to be discovered and elucidated as potential targets for designing effective intervention strategies.^22^, ^23^, ^24^, ^25^

Recently, several groups identified ubiquitously transcribed tetratricopeptide repeat on chromosome X (*UTX*), also known as KDM6A, as a novel histone demethylase that catalyzes the removal of dimethyl and trimethyl groups from histone H3 lysine 27, thereby promoting target gene activation.^27^^,^^28^ Researchers also recently demonstrated a histone demethylation-independent role for UTX in normal and malignant T cells, mesoderm differentiation, and mouse embryonic development.^29^, ^30^, ^31^ Investigators identified somatic loss-of-function mutations of the *UTX* gene in a variety of human tumors, including multiple myeloma, esophageal cancer, and renal cancer.^32^ The identification of recurrent inactivating *UTX* mutations in several leukemia and solid tumor cases strongly supports that UTX acts as a tumor suppressor for human cancers.^33^, ^34^, ^35^ However, the roles of UTX in cancer development and progression appear to be more complicated than initially expected. Two independent studies demonstrated that UTX controls cell-cycle progression via the retinoblastoma protein pathway.^36^^,^^37^ UTX also may function as a bona fide tumor suppressor for T-cell acute lymphoblastic leukemia.^35^ In renal cell carcinoma cases, low expression of UTX is associated with reduced overall survival durations.^38^ In contrast, in patients with breast cancer, high levels of UTX expression are associated with poor prognosis, and UTX knockdown results in significant decreases in the proliferation and invasiveness of breast cancer cells *in vitro* and *in vivo*.^39^

The regulation of UTX expression remains unclear. Previous studies have shown estrogen receptors might be the upstream regulators of UTX.^52^ The importance of GATA6 in gene expression and regulation has recently been reported in many human cancers, including lung cancer,^59^ gastric cancer,^60^ and colorectal cancer,^61^ and the downstream targets have been identified in a series of essential signaling pathways during carcinogenesis. However, whether GATA6 regulates UTX is not known. Loss of GATA6 appears to have a critical role in promoting tumorigenesis and epithelial–mesenchymal transition (EMT)-dependent metastasis in PDA.^57^^,^^58^ The role and mechanisms underlying the interaction between GATA6 and UTX in PDA pathogenesis are unclear.

Whole-genome sequencing and copy number-variation analyses of 100 human PDA specimens indicated that UTX was inactivated in 18% of patients.^40^ However, little is known about the potential function of UTX in PDA development and progression and the mechanisms underlying its potentially dysregulated expression in this tumor. Therefore, in the present study, we examined UTX expression in human PDA cells and specimens, evaluated the impact of altered expression of UTX on PDA development and progression, and explored the underlying mechanisms.

## Materials and methods

### Immunohistochemical analysis

Two human pancreatic tissue cohorts were used in this study. The expression of UTX was analyzed in cohort #1 with 84 primary PDA, 84 adjacent pancreatic intraepithelial neoplasia (PanIN), and normal pancreatic tissue specimens (US Biomax). Correlation analysis of GATA6 and UTX protein expression was performed using cohort #2 with 60 PDA tissues. Immunohistochemical analyses of these specimens and xenografts were conducted with anti-GATA6 (R&D), anti-UTX (Bethyl Laboratories), and anti-Ki67 (Santa Cruz Biotechnology) antibodies as described previously.^20^, ^21^, ^22^, ^23^

### Cell lines

The human embryonic kidney cell line 293T, pancreatic ductal cell line HPNE, and human PDA cell lines AsPC-1, BxPC-3, CaPan-1, CaPan-2, Mia-PaCa-2, PANC-1, and Patu8902 were purchased from the ATCC. The PDA cell lines MDA28 and MDA48 were gifts from Dr. Paul J. Chiao (The University of Texas MD Anderson Cancer Center). The human PDA cell line FG was obtained from Dr. Michael P. Vezeridis (The Warren Alpert Medical School of Brown University).^41^ All these cell lines were maintained in plastic flasks as adherent monolayers in Eagle's minimal essential medium supplemented with 10% FBS, sodium pyruvate, nonessential amino acids, l-glutamine, and a vitamin solution (Flow Laboratories). The ATCC performs characterization and authentication of the cell lines it provides using short tandem repeat profiling, and the cell lines they provided were passaged in our laboratory for fewer than 6 months after reception.^22^, ^23^, ^24^, ^25^

### Immunofluorescent staining

PDA cells were seeded on chamber slides overnight prior to experimentation. Cells were fixed with 4% paraformaldehyde and permeabilized in 0.1% Triton X-100 in PBS and sequentially blocked with 3% bovine serum albumin for 30 min. Following overnight incubation with primary antibodies against UTX, hemagglutinin (HA), or tubulin, the cells were further incubated with appropriate secondary antibodies and subjected to staining.^42^

### Plasmids and small interfering RNAs

The plasmid *pCMV-HA-UTX* was generated by Addgene. Small interfering RNAs (siRNAs) synthesized by Invitrogen were as follows: *UTX* siRNA (#1: 5′-gcaaauguuccaguguauagguuua-3'; stealth#2: 5′-ucaguuagcuuugguugacuguaau-3′) and GATA SiRNA (#1:5′-guggacucuacaugaaacutt-3’; #2, 5′-gcucugguaauagcaauaatt-3′). Negative control siRNA (Invitrogen) and control pCMV vectors were used. Plasmids and siRNAs were transfected into PDA cells using Lipofectamine 2000 CD transfection reagent (Invitrogen). For transient transfection, cells were transfected with plasmids or siRNA at different doses as indicated for 48 h before functional assays.^25^^,^^42^^,^^43^

### Western blot analysis

Standard Western blotting assays were carried out using whole-cell protein lysates; primary antibodies against UTX (Bethyl Laboratories), GATA6 (R&D), H3 (Cell Signaling Technology), HA (Thermo Fisher Scientific), matrix metalloproteinase 2 (MMP2; Santa Cruz Biotechnology), urokinase-type plasminogen activator receptor (uPAR; Santa Cruz Biotechnology), p21 (Santa Cruz Biotechnology), p27 (Santa Cruz Biotechnology), cyclinB1 (Santa Cruz Biotechnology), andcyclinD1 (Santa Cruz Biotechnology); and a secondary antibody (anti-rabbit IgG or anti-mouse IgG; Santa Cruz Biotechnology). Equal protein sample loading was monitored using an anti-α-tubulin, anti-actin, or anti-GAPDH antibody.^24^^,^^25^

### Nuclear and cytoplasmic protein extraction

The NE-PER Nuclear and Cytoplasmic Extraction Reagents (Thermo Fisher Scientific) were used to separate and prepare cytoplasmic and nuclear extracts of 293T and PANC-1 cells, and standard Western blotting was carried out to analyze the protein lysates.^43^

### Animals

Mice were purchased from the Hunan SJA Laboratory Animal CO., LTD (Changsha, Hunan, China). The animal experiments were carried out in strict accordance with the recommendations in the Guide for the Care and Use of Laboratory Animals of the South China University of Technology. The animal protocol was approved by the Committee on Ethics of Animal Experiments of the South China University of Technology.^22^, ^23^, ^24^, ^25^

### Tumor growth and metastasis

Tumor PDA cells (1 × 10^6^) in 0.1 mL of Hank’s balanced salt solution were injected subcutaneously into the right scapular regions of nude mice. The resulting tumors were measured every week. Tumor-bearing mice were killed when they became moribund or on day 28 after injection and their tumors were removed and weighed.^22^, ^23^, ^24^, ^25^ To measure liver metastasis, 1 × 10^6^ tumor cells were injected intravenously into another group of mice via the ileocolic vein. The mice were killed on day 28 and their liver surface metastases were counted.

### Tumor-cell migration/invasion assay

Both cell scratch-wound (horizontal migration) and modified Boyden chamber (vertical invasion) assays were performed to determine the migratory ability and invasiveness of PDA cells with altered UTX expression as described previously.^44^ For the cell scratch-wound assay, cells were grown in six-well plates until confluent. A wound was generated on the surface of the resulting cell monolayer via scraping with the 10-μL tip of a pipette, the cells in the wounded monolayer were photographed at different time points, and cell migration was assessed by measuring gap sizes in multiple fields. For the Boyden chamber assay, 24-well tissue culture plates with 12 cell culture inserts (Millipore) were used. Each insert contained an 8-μm-pore-size polycarbonate membrane with a precoated thin layer of a basement membrane matrix (ECMatrix**;** for the invasion assay) or without a coated matrix (for the migration assay). Ten percent FBS-containing medium was placed in the lower chambers to act as a chemoattractant. Cells (5 × 10^5^) in a 300-μL volume of serum-free medium were placed in the upper chambers and incubated at 37 °C for 48 h. Cells on the lower surface of the polycarbonate membrane, which had invaded the ECMatrix and migrated through the membrane, were stained, counted, and photographed under a microscope.^22^^,^^24^^,^^25^

### Polymerase chain reaction analysis

Polymerase chain reaction (PCR) analysis of genomic UTX was performed using total genomic DNA obtained from tumor cells. Total DNAs were purified using a QIAamp DNA Mini Kit (QIAGEN). The PCR primer sequences were as follows: *UTX* exon18, 5′-tacctcaggtggacaacaagg-3' (forward) and 5′- gcagatctgttttcatgggg-3' (reverse); *UTX* exon1, 5′-gttgtgaattcgctgcgttt-3' (forward) and 5′-tgccttaccttgcccagtag-3' (reverse); and *actin*, 5′-cctgcagagttccaaaggag-3' (forward) and 5′-ggcatcctcaccctgaagta-3' (reverse).^25^^,^^42^^,^^43^

### Reverse transcription-PCR

Reverse transcription-PCR analysis of *UTX* mRNA expression in tumor cells was performed using total RNA. Total RNAs were purified using an RNeasy Plus Mini Kit (QIAGEN), and cDNAs were synthesized using an iScript cDNA Synthesis Kit (Bio-Rad). The PCR primer sequences were as follows: *UTX* exon18, 5′-taaccgcacaaacctgacca-3' (forward) and 5′-tgccttgttgtccacctgag-3' (reverse); *UTX* exon30, 5′-tgtcagacattgagggaagc-3' (forward) and 5′-cggatggtaatggaggagct-3' (reverse); and *GAPDH*, 5′-tgcaccaccaactgcttagc-3' (forward) and 5′-ggcatggactgtggtcatgag-3' (reverse).^24^^,^^42^

### Bioinformatic analysis

The Ualcan (http://ualcan.path.uab.edu/) and LinkedOmics database (http://www.linkedomics.org/) were used as web-based platforms for analyzing TCGA cancer-associated multi-dimensional datasets. Both these websites allowed a flexible exploration of associations between the molecular or clinical attributes of interest. GEO databases (Accession Number GSE71729 and GSE6629) were used to analyze gene expression in PDA. Putative binding sequences of GATA6 in *UTX* promoter were obtained from JASPAR (http://jaspar.genereg.net/) and The Animal Transcription Factor Data Base (Animal TFDB) (http://bioinfo.life.hust.edu.cn/AnimalTFDB/#!/).

### Promoter reporter and dual luciferase assay

PDA cells plated in 96-well plates were transfected with *UTX* promoter reporters, siRNAs, or specific gene expression plasmids using Lipofectamine 2000 (Invitrogen). The *UTX* promoter activity in these cells was normalized via co-transfection of a β-actin/Renilla luciferase reporter containing a full-length *Renilla luciferase* gene. The Dual-luciferase reporter assay was performed 24 h after transfection and quantified using a dual luciferase assay system (Promega). The promoter mutants were generated using the Q5® site-directed mutagenesis kit (NEB, Singapore) according to the manufacturer's instructions. These constructs of truncated or mutated PiHL promoter were subsequently cloned into the pGL3 vector.

### Chromatin immunoprecipitation assay

PDA cells (2 × 10^6^) were prepared for a chromatin immunoprecipitation (ChIP) assay using a ChIP assay kit (Millipore) according to the manufacturer's protocol. The resulting precipitated DNA specimens were analyzed using PCR to amplify fractions of the UTX promoter. The PCR products were resolved electrophoretically on a 2% agarose gel and visualized using ethidium bromide staining.

### Statistical analysis

The two-tailed Pearson *χ*^2^ test or Fisher exact test was used to determine the significance of differences among covariates. All *in vitro* experiments were performed in triplicate and at least three times. Data were presented either as means ± standard deviation from one representative independent experiment of three with similar results or means ± standard error of the mean from three independent experiments. The significance of the *in vitro* and *in vivo* data was determined using the Student *t*-test (two-tailed) or one-way analysis of variance. In all the tests, *P* values less than 0.05 were considered statistically significant. The SPSS software program (version 17.0; IBM Corporation) was used for statistical analysis.

## Results

### Direct correlation of reduced UTX expression with reduced survival durations in PDA patients

**Figure 1:**
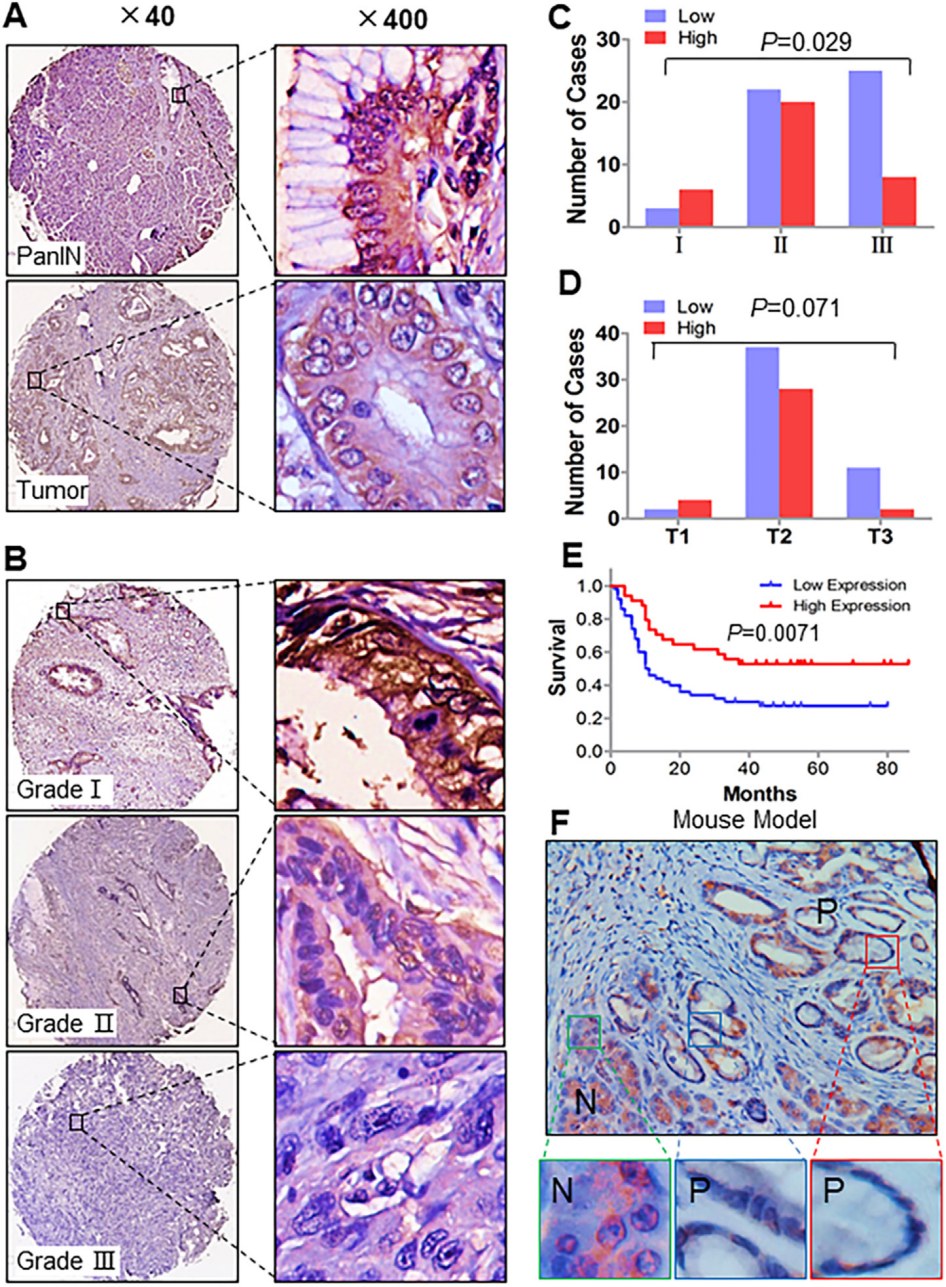
Reduced and lost UTX expression in human PDA specimens. PDA and PanIN specimens in TMAs were immunostained with a specific anti-UTX antibody. **(A)** Representative images of UTX expression in PDA and PanIN specimens. **(B, C)** Positive association of UTX expression with tumor differentiation in PDAs. (**D)** Graphs of the UTX expression in PDA specimens at different T categories. **(E)** Kaplan–Meier analysis of the overall survival times in high and low UTX-expressing PDA patients. **(F)** UTX expression in normal pancreatic tissue (“N”) and PanIN (“P”) specimens obtained from a *Pdx-Cre* mouse with PDA.

To determine the roles of UTX in PDA pathogenesis, we first investigated UTX protein expression in the 84 primary PDA specimens, 84 matched adjacent PanIN specimens, and normal pancreatic tissue specimens in a TMA. The clinicopathologic characteristics of the patients from whom the 84 PDA specimens were obtained are listed in Table S1. We observed decreased expression of UTX in PDA specimens relative to the corresponding PanIN specimens (Fig. 1A). TCGA data showed that UTX expression was higher in pancreatic cancer tissues than that in the normal tissues (Fig. S1A). A similar expression pattern was observed in the colorectal cancer tissues from the TCGA cohort (Fig. S1B). Our immunohistochemical analysis confirmed that UTX protein expression was also attenuated in colorectal cancer tissues relative to the adjacent normal tissues (Fig. S1C). We analyzed the relationship between clinicopathologic parameters and UTX expression in PDA specimens (Table S1). UTX expression was negatively associated with worse histologic differentiation (*P* = 0.029) (Fig. 1B, C) but with advanced T category with statistical significance at borderline (*P* = 0.071) (Fig. 1D). We then investigated the relationship of UTX expression with age, sex, TNM category, and distant metastasis and found no statistically significant differences (*P* > 0.05). Kaplan–Meier analyses demonstrated that expression of UTX was strongly associated with increased overall survival (Fig. 1E). We further examined the UTX expression in the *Pdx-Cre* mouse model of PDA with KRAS mutant. Consistent with our previous finding, UTX expression was higher in normal pancreatic tissue than in PanIN specimens (Fig. 1F). Collectively, UTX expression gradually decreased from normal pancreatic tissue through to pancreatic intraepithelial neoplasia and pancreatic cancer, suggesting that UTX dysregulation is an early event in the multistep progression of pancreatic carcinogenesis.

### Subcellular location of UTX in PDA cells and tissues

To further determine the role of UTX in PDA development and progression, we analyzed the UTX gene integrity in PDA cell lines with a focus on exons and mRNA and protein expression using both 293T and HPNE cells as controls. According to previous findings,^26^ we analyzed the UTX exon18 gene and mRNA expression and protein expression in 293T, HPNE, and PDA cell lines. UTX was highly expressed in 293T, CaPan-2, FG, MDA28, and Patu8902 cells but expressed at relatively low levels in HPNE, AsPC-1, BxPC-3, CaPan-1, MDA48, and PANC-1 cells. Importantly, we found expression of the *UTX* gene, mRNA, and protein was not detectable in Mia-PaCa-2 cells (Fig. S2A–C). To further examine the UTX expression in normal and malignant ductal cells, we primarily cultured PDA cells and normal pancreatic duct epithelial cells from PDA and adjacent normal tissues. Western blot showed that UTX expression was reduced in cancer cells relative to normal cells (data not shown). The general reduction of UTX in PDA may owe to genetic and epigenetic regulation, which may need further exploration. To determine the location of UTX in PDA cells, we first performed immunohistochemical staining of PDA and PanIN specimens for UTX. We found that UTX was localized in both the cytoplasm and nucleus (Fig. S3A). We confirmed this using Western blot analysis of nuclear and cytoplasmic protein extracts from PANC-1 (Fig. S3B) and 293T (Fig. S3C) cells, using H3 and tubulin as nuclear and cytoplasmic control proteins, respectively. Moreover, we confirmed the subcellular localization of UTX using a cell immunofluorescence assay in PANC-1 (Fig. S3D, E) and 293T cells (Fig. S3C).

### Inhibition of PDA cell proliferation and apoptosis *in vitro* and tumor growth *in vivo* by increased UTX expression

**Figure 2:**
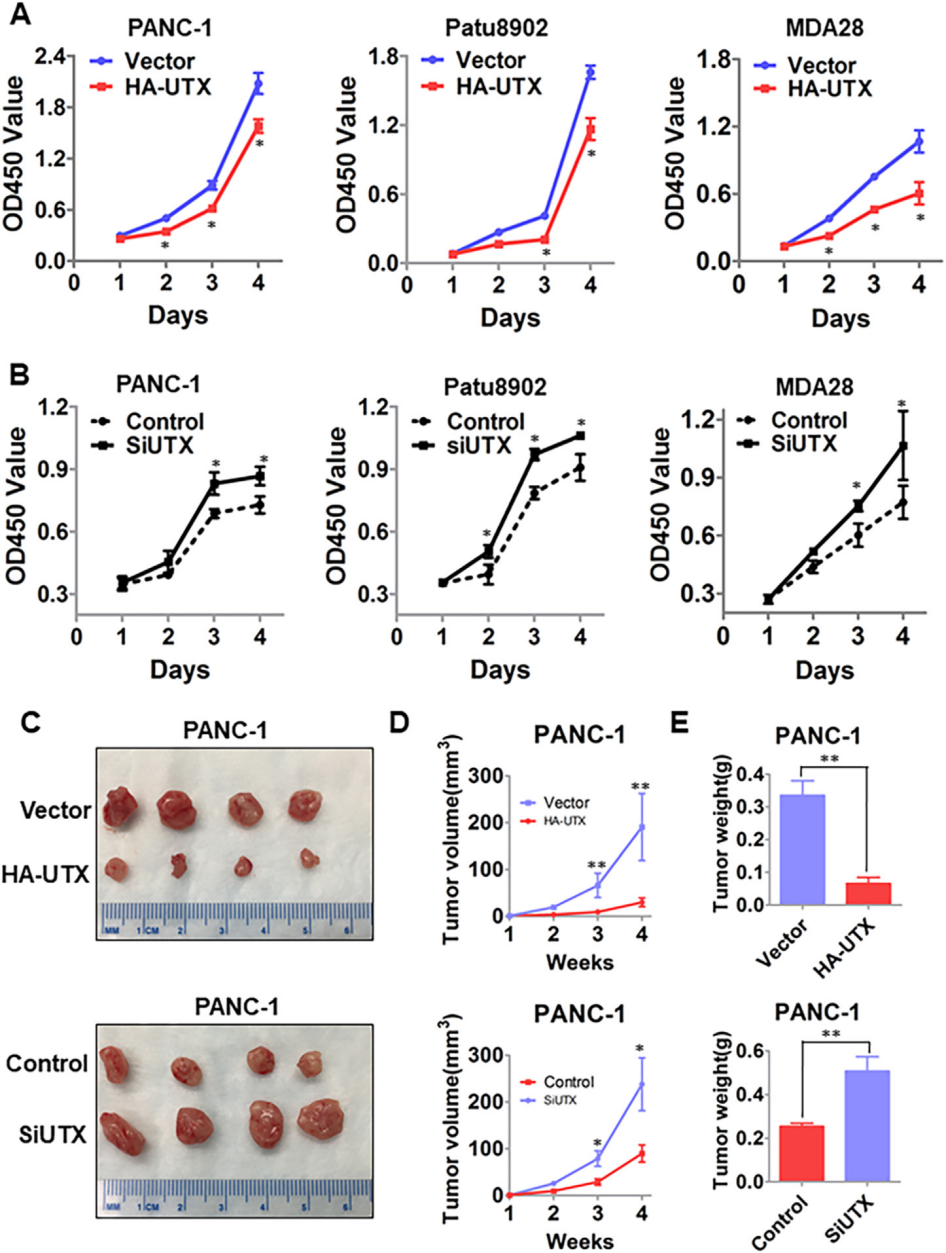
Increased UTX expression suppressed PDA cell proliferation i*n vitro* and *in vivo*. PANC-1, Patu8902, and MDA28 cells were transfected with HA-UTX **(A)** or SiUTX **(B)** and control vectors and siRNAs**,** respectively, for 48 h. The cell growth was assessed using a Cell Counting Kit-8 (according to an optical density at 450 nm [OD_450_]) at the indicated time points. PANC-1 cells with UTX overexpression or knockdown of UTX expression transfected with HA-UTX or SiUTX were injected subcutaneously into the right scapular regions of nude mice (1 × 10^6^ cells per mouse, four mice per group). Gross tumors in the mice **(C)**, tumor growth curves **(D)**, and tumor weights **(E)** are shown. ^∗^*P* < 0.05; ^∗∗^*P* < 0.01.

To determine the effect of UTX on PDA cell proliferation, we induced UTX overexpression via gene transfection and knocked down UTX expression using siRNA (SiUTX#2 with best targeting efficacy) in PANC-1, Patu8902, and MDA28 cells. We found that overexpression of UTX led to decreased PDA cell monolayer growth, whereas knockdown of UTX expression resulted in increased cell growth (Fig. 2A, B). The results of flow cytometric analysis revealed that PDA cells transfected with UTX had an obvious cell cycle arrest in the G0–G1 phase and the population of cells in the S phase was decreased (Fig. S4A). Apoptosis assays revealed that the fraction of apoptotic cells was significantly increased among the UTX-up-regulated PDA cells relative to the control cells (Fig. S4B). In our animal model of PDA, transfection with HA-UTX inhibited the growth of PANC-1 and Patu8902 cells, whereas knockdown of UTX expression promoted the growth of these cells *in vivo* (Fig. 2C–E; Fig. S5A–C). Consistently, immunohistochemical analyses demonstrated that overexpression of UTX led to decreased Ki67 staining, whereas reduced expression of UTX led to increased Ki67 staining (Fig. S5D). These results demonstrated that UTX inhibited the tumor growth of PDA *in vitro* and *in vivo*, supporting that UTX functions as a tumor suppressor in PDA.

### Inhibition of PDA cell migration and invasion *in vitro* and tumor metastasis *in vivo* by increased UTX expression

**Figure 3:**
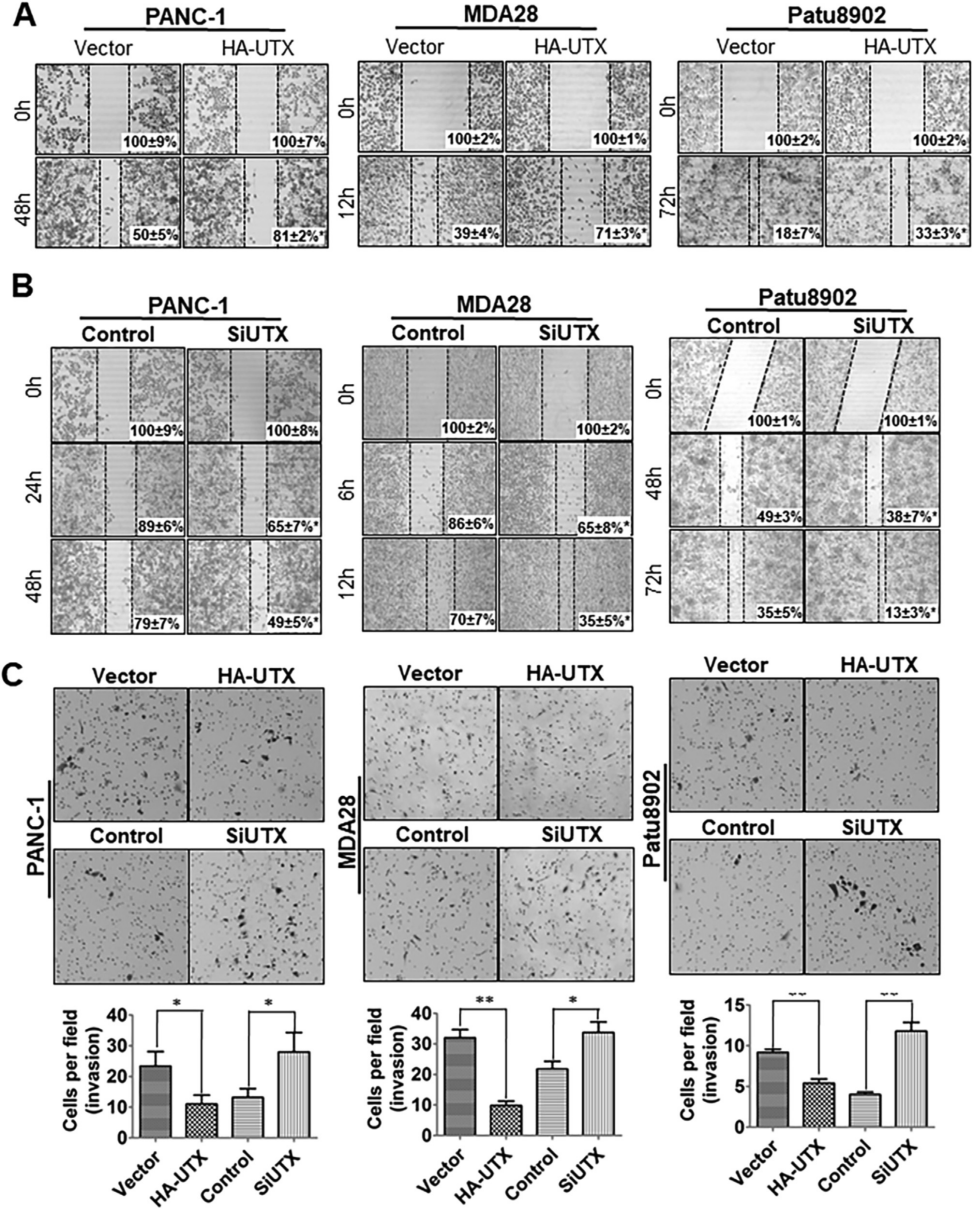
Increased UTX expression suppressed PDA cell migration and invasion *in vitro*. PANC-1, Patu8902, and MDA28 cells were transfected with HA-UTX **(A)** or SiUTX **(B)** and control vectors and siRNAs, respectively, for 48 h. **(A)** For a cell scratch-wound assay, cells in each group were placed in six-well plates, wounded via scratching, and maintained at 37 °C for 12, 24, or 48 h. Cell cultures were photographed, and cell migration was assessed by measuring the cell-free areas in multiple fields (the inset numbers are the mean percentage gap areas ± standard error of the mean from three independent experiments with similar results). **(C)** For an invasion assay, PANC-1, Patu8902, and MDA28 cells were transfected with HA-UTX or SiUTX and control vectors and siRNAs**,** respectively, for 48 h. Invading cells were examined as described in *Materials and methods*. The data represent the means ± standard error of the mean from three independent experiments with similar results. ^∗^*P* < 0.05 in comparisons of the HA-UTX- and SiUTX-treated groups with the control groups (Student *t*-test); ^∗∗^*P* < 0.01 in comparisons of the HA-UTX- and SiUTX-treated groups with the control groups (Student *t*-test).

To determine the effect of UTX expression on PDA migration and invasion, we transfected PANC-1, Patu8902, and MDA28 cells with HA-UTX and UTX SiRNA, respectively, for 48 h. We wounded the monolayer of the transfected cells via scratching and maintained the cells for 6–72 h. These results demonstrated that forced expression of UTX strongly attenuated the flattening and spreading of PDA cells (Fig. 3A), whereas down-regulation of UTX promoted the flattening and spreading of the cells (Fig. 3B). Boyden chamber assay of invasiveness showed consistent results (Fig. 3C). In our animal model of PDA metastasis, liver surface metastases of HA-UTX-transfected cells were much less than those of control cells, whereas those of SiUTX-transfected cells were markedly enhanced (Fig. S6A). We further investigated several markers of cell growth and metastasis with alerted UTX expression by Western blot. Results showed that overexpression of UTX in PANC-1, Patu8902, and MDA28 cells led to decreased expression of MMP2, uPAR, and cyclinD1 and increased expression of p27, whereas knockdown of UTX expression markedly up-regulated that of MMP2, uPAR, and cyclin D1 and decreased p27 in these cell lines (Fig. S6B). UTX is known as a potent histone demethylase. We further examined whether histone methylation agents would impact UTX biofunctions in PDA cells. Treatment of GSKJ4 HCI, a selective inhibitor of the H3K27 histone demethylase, at least partially rescued the inhibitory effects on the proliferative and invasive capability of PDA cells (data not shown). These data further confirmed the tumor suppressor role of UTX in PDA development and progression.

### Direct correlation between GATA6 and UTX expression in PDA cells

**Figure 4:**
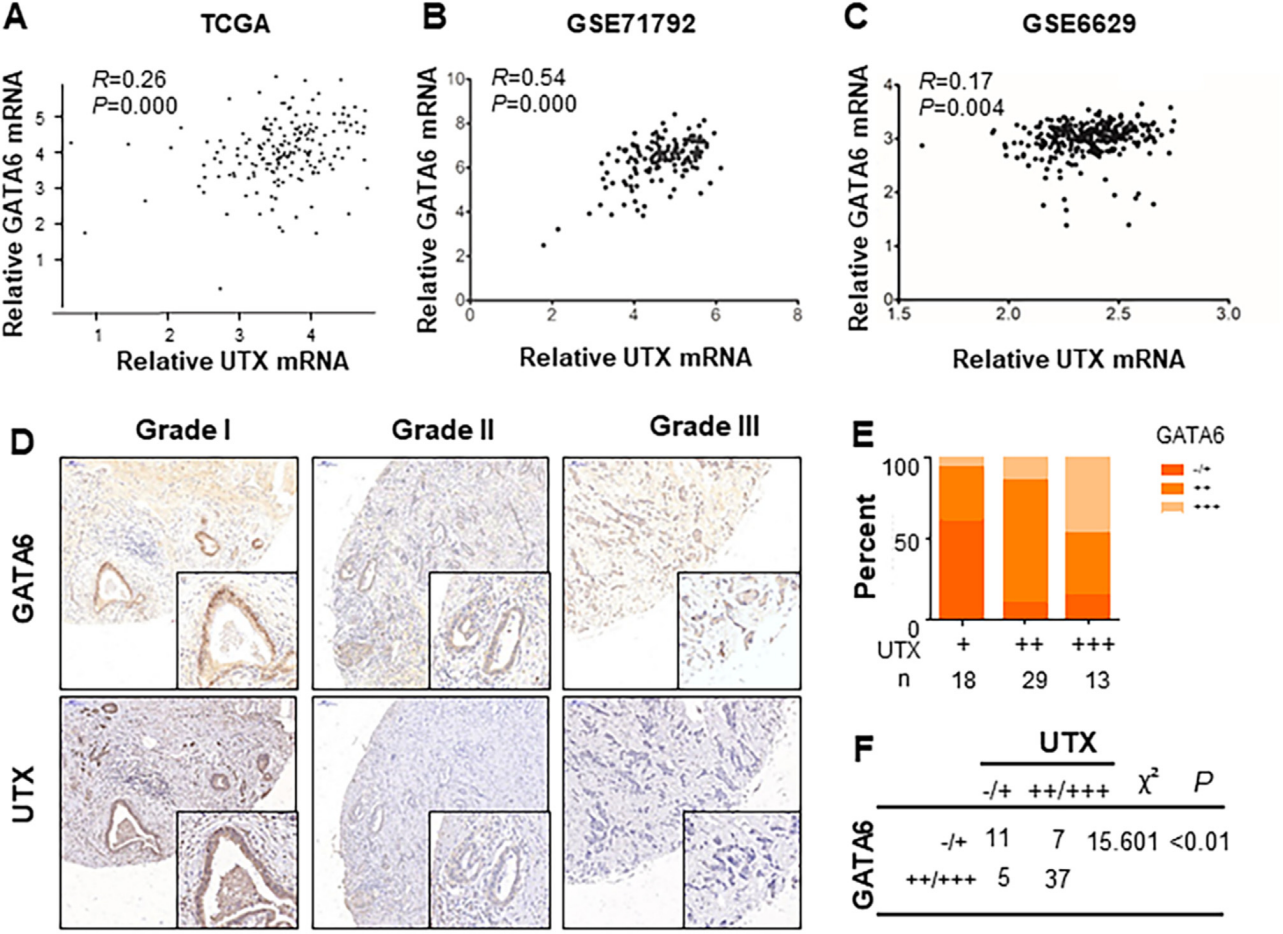
Co-expression of UTX and GATA6 in PDA tissues. **(A)** The correlation analysis between UTX and GATA6 mRNA level made in pancreatic cancer from TCGA database (www.LinkedOmics.org). **(B, C)** Data from the GEO database (GSE71729 and GSE6629) illustrated that GATA6 was positively correlated with UTX mRNA expression. **(D)** Immunohistochemical analysis of GATA6 and UTX correlative expression in human PDA surgical samples (magnification,×200 and ×400 in the inserts). **(E)** The distribution of immunohistochemical results between GATA6 and UTX expression. **(F)** Statistical analysis of immunohistochemical results of GATA6 and UTX expression in human PDA surgical samples.

**Figure 5:**
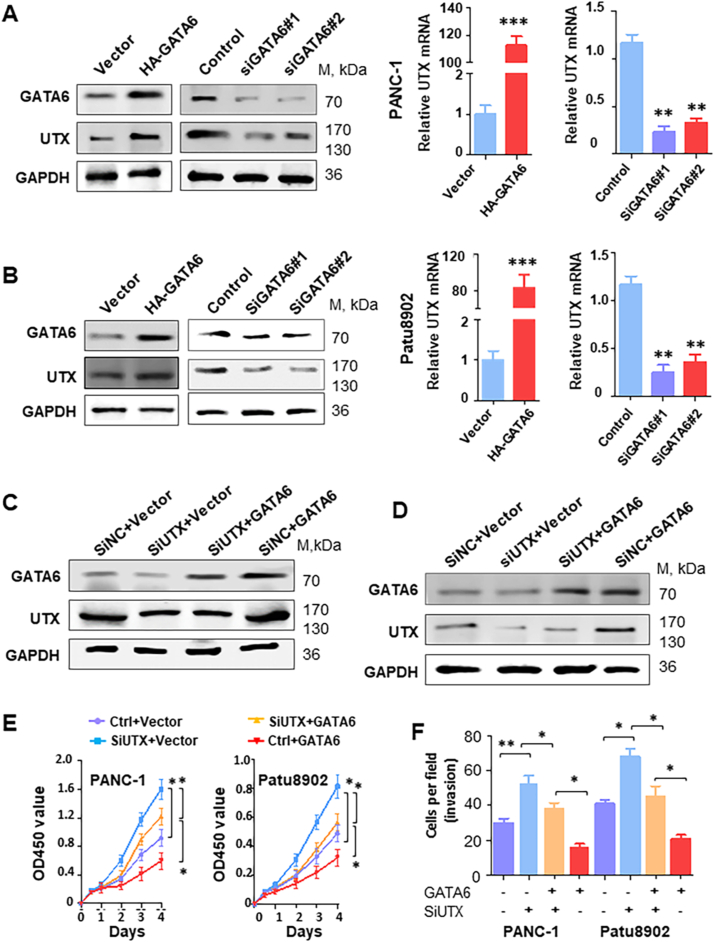
Promotion of UTX expression by GATA6 in PDA cells. PANC-1 and Patu8902 cells were transfected with GATA6 plasmid, control vector, SiGATA6 (#1 and #2), or control siRNA for 48 h. **(A, B)** Total protein and mRNA lysates of PANC-1 (A) and Patu8902 (B) were harvested, and the expression of STK33 and KLF4 in the lysates was determined using real-time Western blotting and PCR. **(C, D)** Western blotting analysis of PANC-1 (C) and Patu8902 (D) cells co-transfected with SiUTX #2 (50 nmol/L) or GATA6 plasmid (2 μg). **(E, F)** CCK8 (E) and Boyden chamber (F) analysis of PANC-1 and Patu8902 cells co-transfected with SiUTX #2 (50 nmol/L) or GATA6 plasmid (2 μg). The data are presented as the means ± SEM from three independent experiments. ^∗^*P* < 0.05.

To identify the regulatory mechanism of UTX as a tumor suppressor during pancreatic tumorigenesis, we made a promoter analysis to find its transcription activators. By using online tools JASPAR and Animal TFDB3.0, databases for the prediction of transcription factor binding sites, we found several potent transcription regulators, including GATA6 (Fig. S7A, B). Our unpublished work demonstrated that GATA6 is a crucial suppressor for the pro-tumor immune microenvironment. GATA6 mRNA expression in PDA tissues may not accurately reflect clinical characteristics. However, its expression in the ductal compartment seems to be correlated with prolonged overall survival (data not shown). To test whether UTX acted as a transcriptional target of GATA6, a series of experiments were performed. First, by using TCGA and GEO databases, we demonstrated that mRNA expression of GATA6 and UTX in PDA were closely correlated (Fig. 4A–C). Next, we examined the protein expression of GATA6 and UTX in another cohort with 30 PDA tissues by immunohistochemical analysis. As shown in Figure 4D, UTX expression co-localized with GATA6 expression in consecutive sections of PDA tissues with different differentiation grades. Importantly, GATA6 expression in PDA specimens was highly positively correlated with UTX expression (Fig. 4E, F). Furthermore, we examined the expression of UTX and UTX-mediated tumor cell aggressiveness with alerted GATA6 expression. PANC-1 cells transfected with GATA6 showed significantly elevated UTX protein and mRNA expression, while cells treated with two GATA6 specific siRNA (#1 and #2) did the opposite (Fig. 5A). Treatment in Patu8902 cells showed consistent results (Fig. 5B). To further understand the role of GATA6 in UTX-mediated PDA aggressiveness, we induced UTX knockdown in PANC-1 and Patu8902 cells with ectopic expression of GATA6 (Fig. 5C, D). Silenced UTX expression partially inhibited the GATA6-induced inhibitory effect on cell proliferation (Fig. 5E) and invasion (Fig. 5F) of PDA cells. These results clearly indicated that GATA6 up-regulated UTX expression and that UTX is an important mediator of GATA6-induced PDA progression.

### Transcriptional activation of UTX expression by GATA6

**Figure 6:**
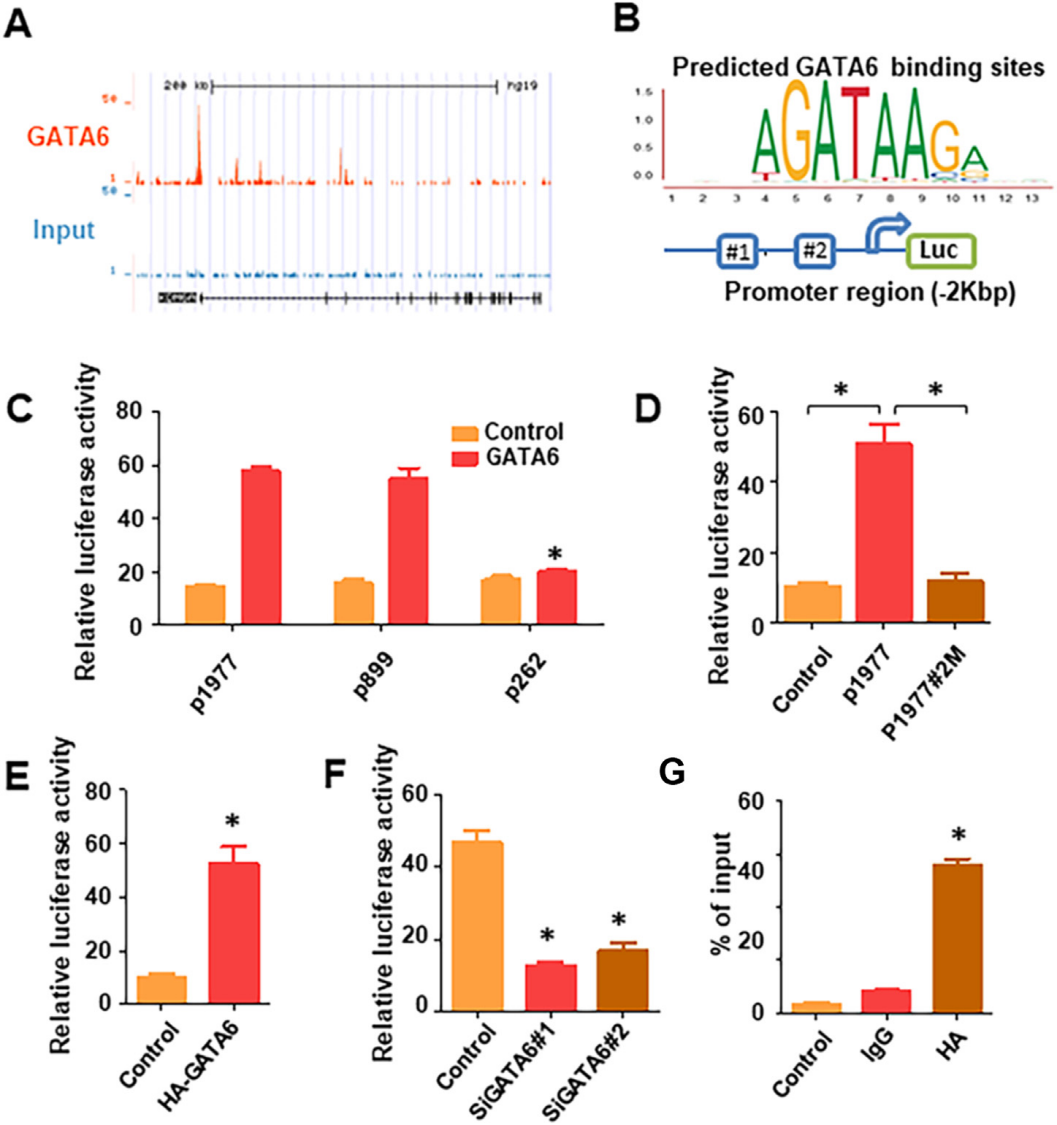
Direct activation of UTX transcription by GATA6. (**A**) Representation of ChIP-Seq peaks on UTX promoters. The online data portal for ChIP-Seq data, Cistrome Data Browser (cistrome.org), was used to test the possibility of GATA6 binding to the UTX promoter. **(B)** Two GATA6-binding sites located at different sites in the UTX promoter sequence. **(C)** UTX promoter reporters p1977, p899, and p262 were transfected into 239T cells in triplicate with GATA6 expression or control vectors for 24 h. The UTX promoter activity was then examined using a dual luciferase assay kit. **(D)** Luciferase-based reporter assay showing the activity of UTX promoter reporters in 293T cells co-transfected with GATA6 plasmid and empty vector or with vectors expressing either wild-type or GATA6-binding site#2 mutated UTX promoter. **(E)** PANC-1 cells were co-transfected with p1799, GATA6 plasmid, or control vector. **(F)** PANC-1 cells were co-transfected with p1799, SiGATA6 (#1 or #2), or a non-targeting siRNA. The promoter activities in the cells determined using a dual luciferase assay are shown. **(G)** The results of the ChIP assay conducted using chromatins isolated from PANC-1 cells. The immunoprecipitated DNA was analyzed by PCR followed by agarose gel electrophoresis. Genomic DNA input was 1%. The experiments were performed three times independently. ^∗^*P* < 0.05.

To determine whether GATA6 transcriptionally activates UTX expression, we first explored Cistrome Data Browser (cistrome.org), an online data portal for ChIP-Seq data. Prominent GATA6 peaks in UTX suggested strong binding (Fig. 6A). Next, we analyzed the UTX promoter sequence for the presence of potential GATA6 binding sites containing (A/T) GATA (A/G) motif (Fig. 6B) and identified two putative GATA6-binding elements (referred to as GBE#1 and #2). Accordingly, we constructed the deletion mutant reporters p1799 (containing GBE#1 and #2), p899 (containing GBE#2), and p262 (containing no GBE). We co-transfected the deletion mutant reporters with or without GATA6 expression vectors into 293T cells. Luciferase reporter assay results demonstrated that deletion of the region covering binding site#2 markedly decreased the promoter activity of UTX activated by GATA6 (Fig. 6C). In addition, we determined the activity of wide-type promoter, instead of GBE#2 mutant one, got promoted with enforced GATA6 expression (Fig. 6D). To further determine whether GATA6 regulates UTX promoter transcriptional activity in PDA cells, we co-transfected a full-length reporter with GATA6 expression vectors or siRNA into PDA cells. As shown in Figure 6E and F, increased GATA6 expression in PANC-1 cells induced UTX promoter activity, whereas knockdown of GATA6 expression showed an inhibiting effect. A similar result was found in Patu8902 cells (Fig. S8A, B). ChIP assay also confirmed GATA6 directly bound GBE#2 in PDA cells (Fig. 6J; Fig. S8C). Taken together, these results suggested that GATA6 can not only directly bind to the UTX promoter but also influence the biofunctions of UTX in PDA.

## Discussion

In this study, we determined the role of UTX expression and its regulatory mechanism during PDA development and progression. We found that UTX expression was drastically reduced in human PDA cell lines and specimens. Low UTX expression was associated with worse differentiation and poor prognosis of PDA. Forced UTX expression inhibited PDA proliferation, migration, and invasion *in vitro* and growth and metastasis *in vivo*, whereas knockdown of UTX expression did the opposite. UTX attenuated PDA cell aggressiveness by serving as an important target of transcriptional factor GATA6, as demonstrated in PDA cell lines, the orthotopic mouse model, and human PDA specimens. Mechanistically, GATA6 bound directly to the promoter region of UTX and activated its transcriptional activity. Our clinical and mechanistic data has established that UTX is a tumor suppressor in PDA and GATA6-UTX axis may serve as a potential target for PDA therapy.

The conflicting role of UTX in cancer gains increasing attention recently. Low expression of UTX is associated with reduced overall and disease-free survival durations in clear cell renal cell carcinoma patients and appears to be an independent prognostic factor.^38^ A similar result is reported in glioblastoma.^45^ However, in a study of estrogen receptor-positive breast cancer, no clinical parameters are significantly associated with UTX expression.^46^ These discrepancies suggest the complex role of UTX in tumor progression, likely stage- and context-dependent. In the present study, we delineated the relationship between UTX expression in PanIN and PDA and the patients' clinicopathologic characteristics. We found that reduced UTX expression was associated with poor tumor differentiation. Decreased UTX expression was directly associated with reduced overall survival time in the PDA patients. Consistently, PDA mouse models produced similar results. Interestingly, UTX was localized in both nuclei and cytoplasm of PDA cells. However, the significance and potential mechanisms of the subcellular localization of UTX protein and its translocation to the cytoplasm and nucleus remain unclear, warranting further study.

*UTX* is frequently mutated in myeloma cells, and overall survival durations are shorter in myeloma patients with a *UTX* mutation or deletion than in those without mutation.^47^ A recent study has shown that *UTX* is inactivated in 18% of PDA patients (eight homozygous deletions, five structural variants, four frame shifts, two missense mutations, and one in-frame deletion).^40^ In most cases with inactivated UTX, both alleles of *UTX* are affected. In the present study, we determined the UTX expression pattern in nine PDA cell lines and found that expression of UTX mRNA and protein was substantially reduced, while a genetic loss of *UTX* occurred only in one cell line, suggesting that down-regulation of *UTX* gene is mostly through an epigenetic mechanism.

UTX exhibits pro-tumor and anti-tumor activities.^49^, ^50^, ^51^, ^52^ However, a systematic screen by using CRISPR/Cas9-mediated somatic gene knockout in a *Kras*^*G12D/+*^mouse model has confirmed that loss of UTX significantly promotes lung tumorigenesis.^50^ UTX mutant cells show an increased *in vitro* and *in vivo* sensitivity to inhibition of EZH2, a histone methyltransferase that generates H3K27me3.^53^ UTX deficiency renders lymphoma sensitive to cytarabine treatment.^54^ UTX could restrain *Kras*^*G12D*^-driven PDA and confers its sensitivity to BET inhibitors.^55^ In our biologic studies, knockdown of UTX expression markedly enhanced PDA tumor growth and metastasis *in vitro* and *in vivo*, whereas increased UTX did the opposite. All these findings consistently and strongly support that UTX functions as a tumor suppressor gene.

UTX was identified as X chromosome-encoded histone demethylase. Therefore, its roles in regulating methylation and demethylation in tumor cells are widely investigated. UTX could directly influence the methylation level or indirectly through targeting other epigenetic regulators. Knockout of UTX increases the EZH2 level, thus up-regulating the H3K27me3 level. UTX could coordinate with JHDM1D and CBP to direct the H3K27 methylation–acetylation transition and to create a permissive chromatin state on ER targets in breast cancer.^52^ The most significant network of genes bound by UTX is tied to cell-cycle pathways centered on the tumor suppressor gene retinoblastoma and retinoblastoma-binding proteins.^36^ A similar finding is made in a genetic screen of *Drosophila* for ectopic cell growth mutants.^37^ However, UTX appears to play an oncogenic role in breast cancer and TAL1-driven T-cell acute lymphoblastic leukemia.^39^^,^^48^ Estrogen receptor-induced UTX could activate ER expression, forming a feed-forward loop in the regulation of hormone response of breast cancer cells.^52^ Despite the wide mechanism exploration of UTX in other types of cancer, understanding the role of UTX in PDA development and progression is limited. UTX loss could deregulate the COMPASS-like complex and aberrant activation of super-enhancers regulating *ΔNp63*, *MYC*, and *RUNX3* oncogenes selectively in female patients.^55^ However, the mechanism of aberrant loss of *UTX* during pancreatic tumorigenesis is still unknown.

Recent studies have identified GATA6 as a transcriptional factor and tumor suppressor in a variety of human cancers, including PDA. GATA6 contributes to complete pancreatic acinar differentiation and maintains its exocrine function.^56^ *In vivo* evidence from genetically engineered mouse models shows that acinar GATA6 suppresses *Kras*^*G12V*^-driven pancreatic tumorigenesis.^57^ GATA6 is also critical for canonical pancreatic epithelial differentiation. Loss of GATA6 promotes epithelial–mesenchymal transition and metastasis.^58^ Given the important role of GATA6 during pancreatic differentiation and malignancy transition, key downstream target genes and signaling pathways warrant further exploration. In the current study, we provided several lines of evidence that UTX is a direct target of GATA6 in PDA and that GATA6-UTX signaling is critical in PDA progression.

In summary, UTX is a potential biomarker for PDA and functions as a tumor suppressor gene, and inhibits PDA growth and metastasis. Mechanistically, GATA6 activates UTX expression by direct binding to its promoter region and activating its transcription. Thus, the UTX-GATA6 signaling axis offers a potentially novel therapeutic target for PDA treatment.

## Conflict of interests

The authors declare no conflict of interests.

## Funding

This work was supported by the Jiangxi Science Fund for Distinguished Young Scholars (China) (No. 20212ACB216012), the Funding Program for Academic and Technical Leaders of Main Subjects in Jiangxi Province, China (No. 20213BCJ22009 to H.Q. Zhang), and the 10.13039/100014717National Natural Science Foundation of China (No. 81460372 to H.Q. Zhang, No. 81960528 to S. Zheng), the 10.13039/501100008111Hainan Province Science and Technology special fund (China) (ZDYF2020132 to S. Zheng) , the Innovation Platform for Academicians of Hainan Province (China) (YSPTZX202208 to S. Zheng), and Hainan Province Clinical Medical Center (QWYH2021276), the Cardiovascular Disease Research Science Innovation Group of Hainan Medical University (China).

## References

[1] Sung H., Ferlay J., Siegel R.L. (2021). Global cancer statistics 2020: GLOBOCAN estimates of incidence and mortality worldwide for 36 cancers in 185 countries. CA A Cancer J Clin.

[2] Siegel R., Miller K., Fuchs H.E. (2022). Cancer statistics, 2022. CA A Cancer J Clin.

[3] Connor A.A., Gallinger S. (2022). Pancreatic cancer evolution and heterogeneity: integrating omics and clinical data. Nat Rev Cancer.

[4] Wood L.D., Canto M.I., Jaffee E.M. (2022). Pancreatic cancer: pathogenesis, screening, diagnosis, and treatment. Gastroenterology.

[5] Klatte D.C.F., Wallace M.B., Löhr M. (2022). Hereditary pancreatic cancer. Best Pract Res Clin Gastroenterol.

[6] Nießen A., Hackert T. (2022). State-of-the-art surgery for pancreatic cancer. Langenbeck's Arch Surg.

[7] Hou J., Li X., Xie K.P. (2021). Coupled liquid biopsy and bioinformatics for pancreatic cancer early detection and precision prognostication. Mol Cancer.

[8] Yu S., Zhang C., Xie K.P. (2021). Therapeutic resistance of pancreatic cancer: roadmap to its reversal. Biochim Biophys Acta Rev Cancer.

[9] Li S., Xie K. (2022). Ductal metaplasia in pancreas. Biochim Biophys Acta Rev Cancer.

[10] Hosoda W., Wood L.D. (2016). Molecular genetics of pancreatic neoplasms. Surg Pathol Clin.

[11] Xie D., Xie K. (2015). Pancreatic cancer stromal biology and therapy. Genes Dis.

[12] Makohon-Moore A., Iacobuzio-Donahue C.A. (2016). Pancreatic cancer biology and genetics from an evolutionary perspective. Nat Rev Cancer.

[13] Xie V.K., Maitra A. (2017). Krüppel-like factor 4 promotes pancreatic acinar-to-ductal *Metaplasia* and tumor initiation. Pancreas.

[14] Stanger B.Z., Hebrok M. (2013). Control of cell identity in pancreas development and regeneration. Gastroenterology.

[15] Hruban R.H., Goggins M., Parsons J. (2000). Progression model for pancreatic cancer. Clin Cancer Res.

[16] Kern S.E. (2000). Molecular genetic alterations in ductal pancreatic adenocarcinomas. Med Clin North Am.

[17] Almoguera C., Shibata D., Forrester K. (1988). Most human carcinomas of the exocrine pancreas contain mutant c-K-*ras* genes. Cell.

[18] Rozenblum E., Schutte M., Goggins M. (1997). Tumor-suppressive pathways in pancreatic carcinoma. Cancer Res.

[19] Korc M. (2003). Pathways for aberrant angiogenesis in pancreatic cancer. Mol Cancer.

[20] Huang C., Du J., Xie K. (2014). FOXM1 and its oncogenic signaling in pancreatic cancer pathogenesis. Biochim Biophys Acta.

[21] Wei D., Wang L., Kanai M. (2010). KLF4α up-regulation promotes cell cycle progression and reduces survival time of patients with pancreatic cancer. Gastroenterology.

[22] Kong F., Sun T., Kong X. (2018). Krüppel-like factor 4 suppresses serine/threonine kinase 33 activation and metastasis of gastric cancer through reversing epithelial-mesenchymal transition. Clin Cancer Res.

[23] Huang C., Qiu Z., Wang L. (2012). A novel FoxM1-caveolin signaling pathway promotes pancreatic cancer invasion and metastasis. Cancer Res.

[24] Wei D., Wang L., Yan Y. (2016). KLF4 is essential for induction of cellular identity change and acinar-to-ductal reprogramming during early pancreatic carcinogenesis. Cancer Cell.

[25] Li L., Li Z., Kong X. (2014). Down-regulation of microRNA-494 via loss of SMAD4 increases FOXM1 and β-catenin signaling in pancreatic ductal adenocarcinoma cells. Gastroenterology.

[26] van Haaften G., Dalgliesh G.L., Davies H. (2009). Somatic mutations of the histone H3K27 demethylase gene *UTX* in human cancer. Nat Genet.

[27] Lan F., Bayliss P.E., Rinn J.L. (2007). A histone H3 lysine 27 demethylase regulates animal posterior development. Nature.

[28] Agger K., Cloos P.A.C., Christensen J. (2007). UTX and JMJD3 are histone H3K27 demethylases involved in *HOX* gene regulation and development. Nature.

[29] Miller S.A., Mohn S.E., Weinmann A.S. (2010). Jmjd3 and UTX play a demethylase-independent role in chromatin remodeling to regulate T-box family member-dependent gene expression. Mol Cell.

[30] Wang C., Lee J.E., Cho Y.W. (2012). UTX regulates mesoderm differentiation of embryonic stem cells independent of H3K27 demethylase activity. Proc Natl Acad Sci U S A.

[31] Shpargel K.B., Sengoku T., Yokoyama S. (2012). UTX, and UTY demonstrate histone demethylase-independent function in mouse embryonic development. PLoS Genet.

[32] van Haaften G., Dalgliesh G.L., Davies H. (2009). Somatic mutations of the histone H3K27 demethylase gene *UTX* in human cancer. Nat Genet.

[33] Gui Y., Guo G., Huang Y. (2011). Frequent mutations of chromatin remodeling genes in transitional cell carcinoma of the bladder. Nat Genet.

[34] Mar B.G., Bullinger L., Basu E. (2012). Sequencing histone-modifying enzymes identifies UTX mutations in acute lymphoblastic leukemia. Leukemia.

[35] Van der Meulen J., Sanghvi V., Mavrakis K. (2015). The H3K27me3 demethylase UTX is a gender-specific tumor suppressor in T-cell acute lymphoblastic leukemia. Blood.

[36] Wang J.K., Tsai M.C., Poulin G. (2010). The histone demethylase UTX enables RB-dependent cell fate control. Genes Dev.

[37] Herz H.M., Madden L.D., Chen Z. (2010). The H3K27me3 demethylase dUTX is a suppressor of Notch- and Rb-dependent tumors in *Drosophila*. Mol Cell Biol.

[38] Wang J., Liu L., Xi W. (2016). Prognostic value of UTX expression in patients with clear cell renal cell carcinoma. Urol Oncol.

[39] Kim J.H., Sharma A., Dhar S.S. (2014). UTX and MLL4 coordinately regulate transcriptional programs for cell proliferation and invasiveness in breast cancer cells. Cancer Res.

[40] Waddell N., Pajic M., Patch A.M. (2015). Whole genomes redefine the mutational landscape of pancreatic cancer. Nature.

[41] Vezeridis M.P., Tzanakakis G.N., Meitner P.A. (1992). *In vivo* selection of a highly metastatic cell line from a human pancreatic carcinoma in the nude mouse. Cancer.

[42] Zhang N., Wei P., Gong A. (2011). FoxM1 promotes β-catenin nuclear localization and controls Wnt target-gene expression and glioma tumorigenesis. Cancer Cell.

[43] Zhou A., Lin K., Zhang S. (2016). Nuclear GSK3β promotes tumorigenesis by phosphorylating KDM1A and inducing its deubiquitylation by USP22. Nat Cell Biol.

[44] Kong X., Li L., Li Z. (2013). Dysregulated expression of FOXM1 isoforms drives progression of pancreatic cancer. Cancer Res.

[45] Kim J., Lee S.H., Jang J.H. (2017). Increased expression of the histone H3 lysine 4 methyltransferase MLL4 and the histone H3 lysine 27 demethylase UTX prolonging the overall survival of patients with glioblastoma and a methylated MGMT promoter. J Neurosurg.

[46] Bae W.K., Yoo K.H., Lee J.S. (2015). The methyltransferase EZH2 is not required for mammary cancer development, although high EZH2 and low H3K27me3 correlate with poor prognosis of ER-positive breast cancers. Mol Carcinog.

[47] Pawlyn C., Kaiser M.F., Heuck C. (2016). The spectrum and clinical impact of epigenetic modifier mutations in myeloma. Clin Cancer Res.

[48] Benyoucef A., Palii C.G., Wang C. (2016). UTX inhibition as selective epigenetic therapy against TAL1-driven T-cell acute lymphoblastic leukemia. Genes Dev.

[49] Tang X., Cai W., Cheng J. (2019). The histone H3 lysine-27 demethylase UTX plays a critical role in colorectal cancer cell proliferation. Cancer Cell Int.

[50] Wu Q., Tian Y., Zhang J. (2018). *In vivo* CRISPR screening unveils histone demethylase UTX as an important epigenetic regulator in lung tumorigenesis. Proc Natl Acad Sci U S A.

[51] Li S.H., Lu H.I., Huang W.T. (2018). The prognostic significance of histone demethylase UTX in esophageal squamous cell carcinoma. Int J Mol Sci.

[52] Xie G., Liu X., Zhang Y. (2017). UTX promotes hormonally responsive breast carcinogenesis through feed-forward transcription regulation with estrogen receptor. Oncogene.

[53] Ezponda T., Dupéré-Richer D., Will C.M. (2017). UTX/KDM6A loss enhances the malignant phenotype of multiple myeloma and sensitizes cells to EZH2 inhibition. Cell Rep.

[54] Li X., Zhang Y., Zheng L. (2018). UTX is an escape from X-inactivation tumor-suppressor in B cell lymphoma. Nat Commun.

[55] Andricovich J., Perkail S., Kai Y. (2018). Loss of KDM6A activates super-enhancers to induce gender-specific squamous-like pancreatic cancer and confers sensitivity to BET inhibitors. Cancer Cell.

[56] Martinelli P., Cañamero M., del Pozo N. (2013). Gata6 is required for complete acinar differentiation and maintenance of the exocrine pancreas in adult mice. Gut.

[57] Martinelli P., Madriles F., Cañamero M. (2016). The acinar regulator Gata6 suppresses KrasG12V-driven pancreatic tumorigenesis in mice. Gut.

[58] Martinelli P., Pau E.C.D.S., Cox T. (2017). GATA6 regulates EMT and tumour dissemination, and is a marker of response to adjuvant chemotherapy in pancreatic cancer. Gut.

[59] Cheung W.C., Zhao M., Liu Z. (2013). Control of alveolar differentiation by the lineage transcription factors GATA6 and HOPX inhibits lung adenocarcinoma metastasis. Cancer Cell.

[60] Dulak A.M., Schumacher S.E., van Lieshout J. (2012). Gastrointestinal adenocarcinomas of the esophagus, stomach, and colon exhibit distinct patterns of genome instability and oncogenesis. Cancer Res.

[61] Tsuji S., Kawasaki Y., Furukawa S. (2014). The miR-363-GATA6-Lgr5 pathway is critical for colorectal tumourigenesis. Nat Commun.

