# Supplementary material for: Loss of GATA6-mediated up-regulation of UTX promotes pancreatic tumorigenesis and progression
Source: Genes Dis. 2023 Mar 31;11(2):921–34. doi: 10.1016/j.gendis.2023.01.019 (PMC10491869; doi:10.1016/j.gendis.2023.01.019)
Supplement: Multimedia component 1 [file mmc1.pdf]

Supplementary Figures and Legends

Zhang *et al.*, Figure S1

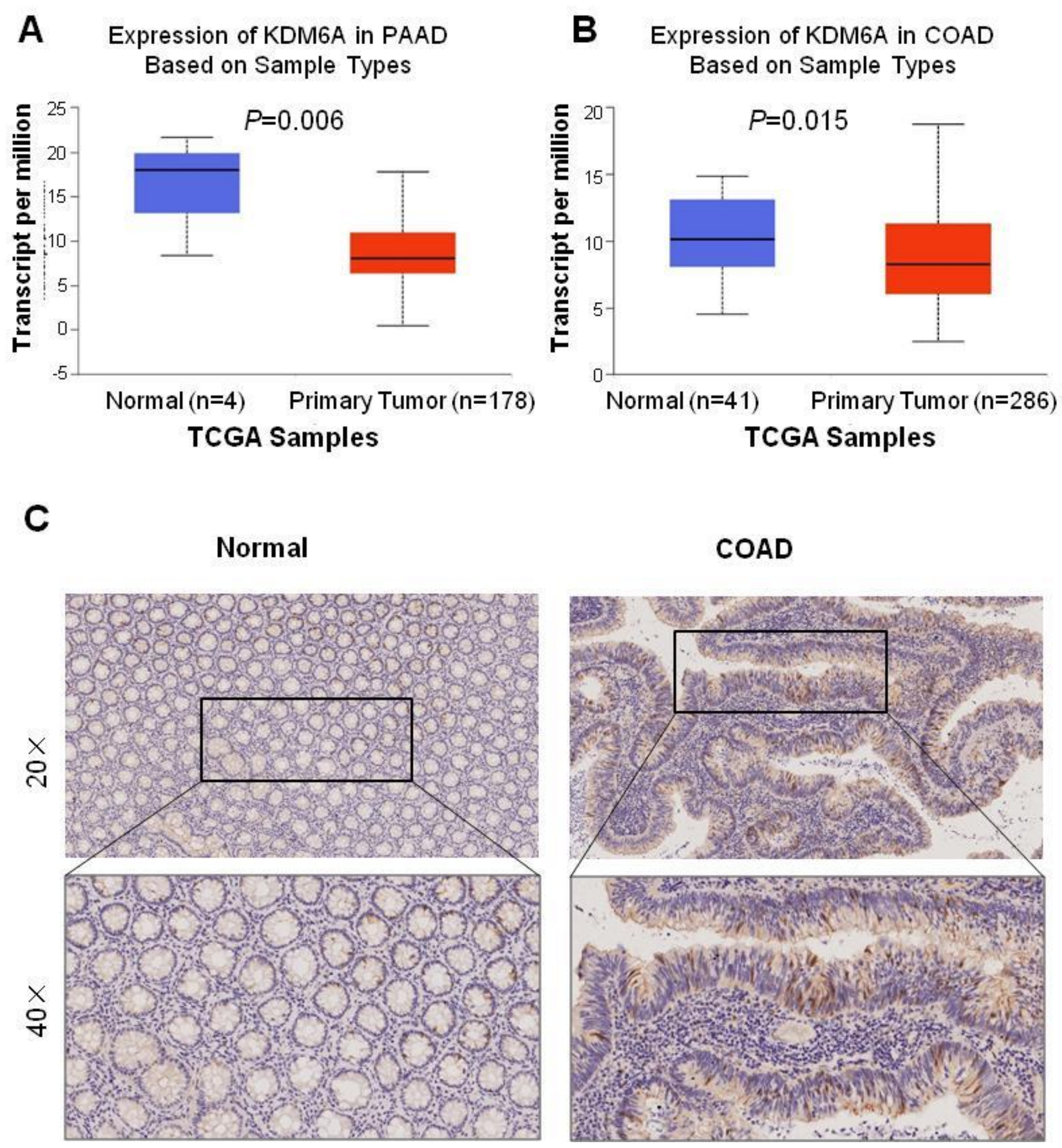

Figure S1. Expression of UTX in cancer. A, UTX expression in PDA tissues and normal pancreatic tissues from TCGA database. **B**, UTX expression in COAD tissues and normal pancreatic tissues from TCGA database. TCGA data was analyzed by using online tool The Ualcan (http://ualcan.path.uab.edu/).**C**, UTX expression in COAD tissues and normal pancreatic tissues by IHC.

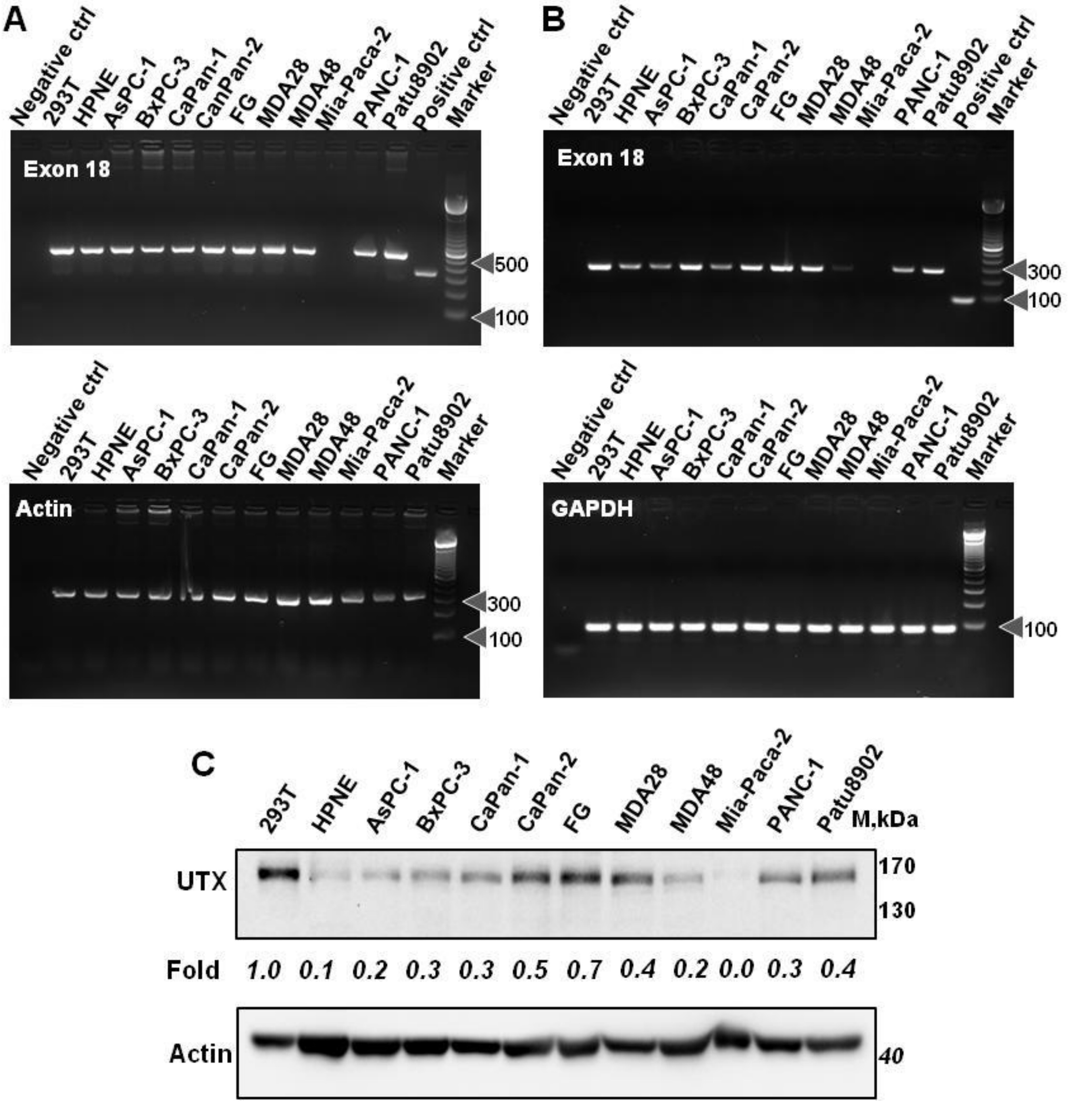

Figure S2. Reduced expression of UTX in PDA cells. A, PCR analysis results verifying the integrity of *UTX* gene expression (primers were designed to target its exon18) in HPNE and 293T cells and PDA cell lines. **B**, reverse transcription-PCR analysis of the expression of UTX mRNA in HPNE and 293T cells and PDA cell lines. **C**, Western blot analysis of UTX protein expression in PDA cell lines, and HPNE and 293T cells.

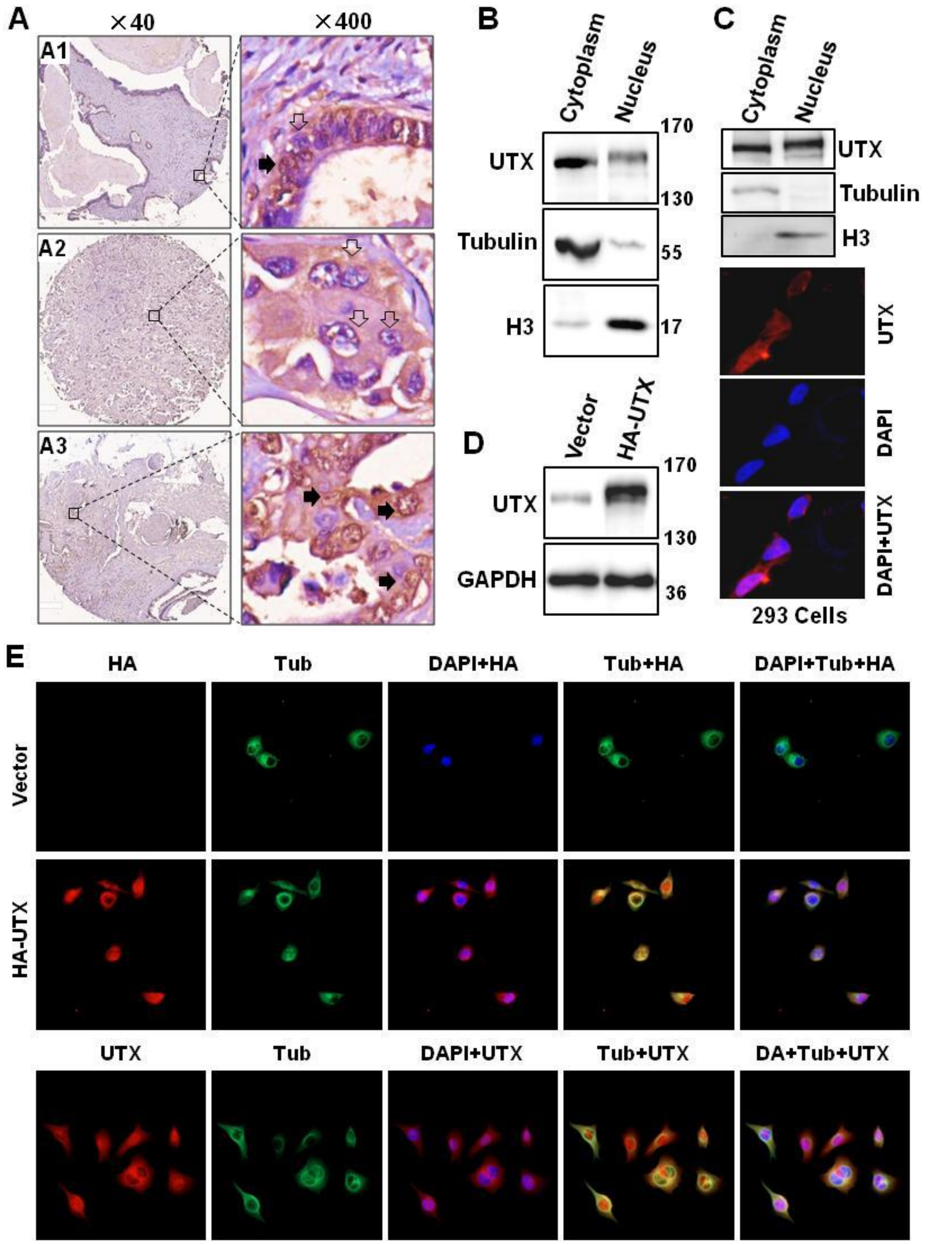

Figure S3. The subcellular localization of UTX protein in PDA specimens and cells. A, Immunohistochemical stains of PDA and PanIN specimens for UTX showing that it was localized in both cytoplasm and nuclei. **B**, Western blot assays showing the expression of UTX protein after extraction of the nuclear and cytoplasmic UTX protein in PANC-1 cells. **C**, Western blot assay and cell immunofluorescence demonstrating that UTX localizes in both cytoplasm and nuclei in 293T cells. **D**, Transfection efficacy of HA-UTX determined by western blot. **E**, Cell immunofluorescence verifying the subcellular location of UTX in PANC-1 cells. Tub, tubulin; DA, DAPI.

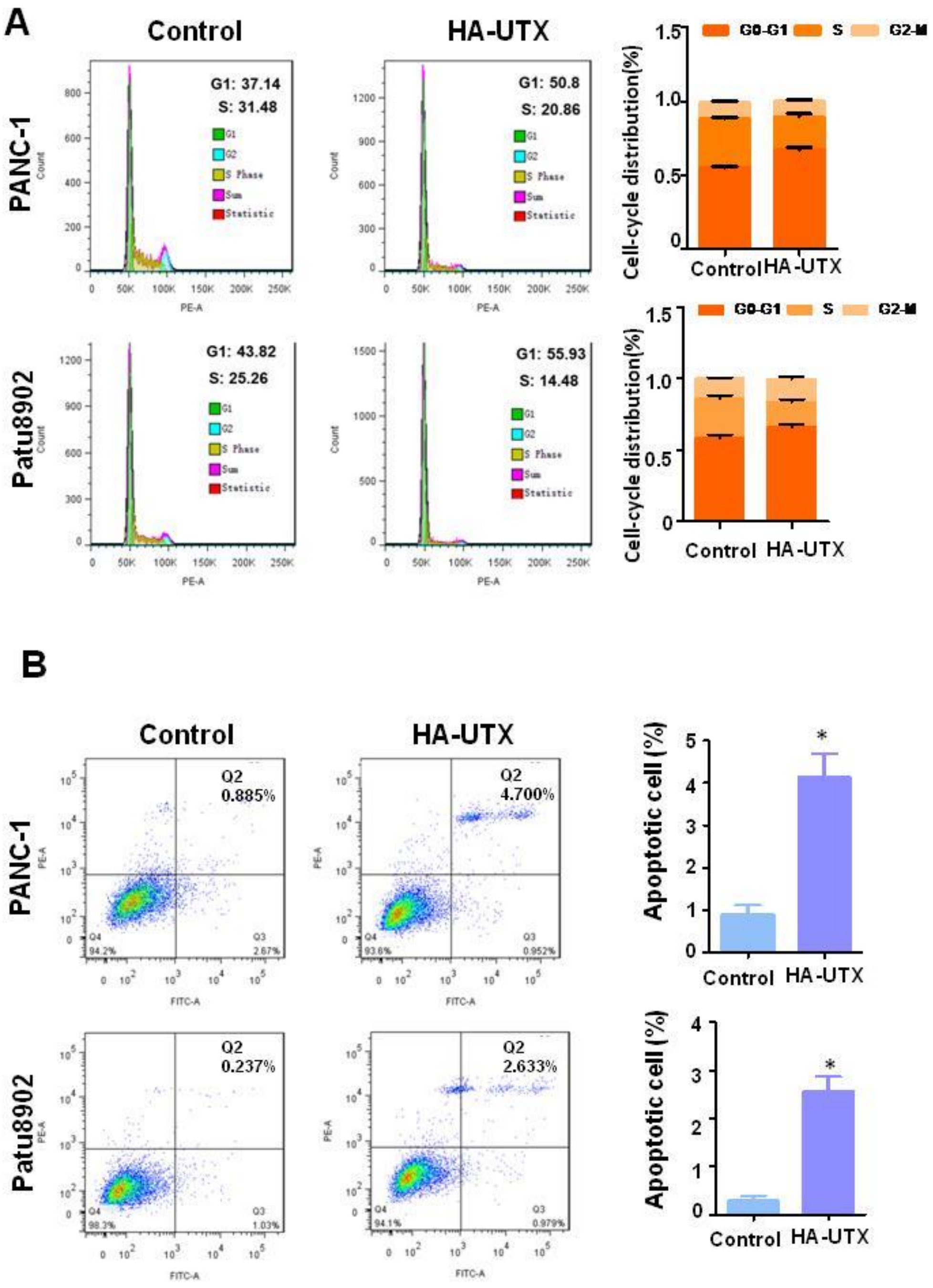

Figure S4. UTX inhibits proliferation and suppresses apoptosis of PDA cells *in vitro*. A, Flow cytometric analysis of cell cycle arrest 48 h after transfection with HA-UTX in PANC-1 and Patu8902 cells. **B**, Flow cytometry analysis of the apoptotic rates in PANC-1 and Patu8902 cells after HA-UTX transfection. *P<0.05.

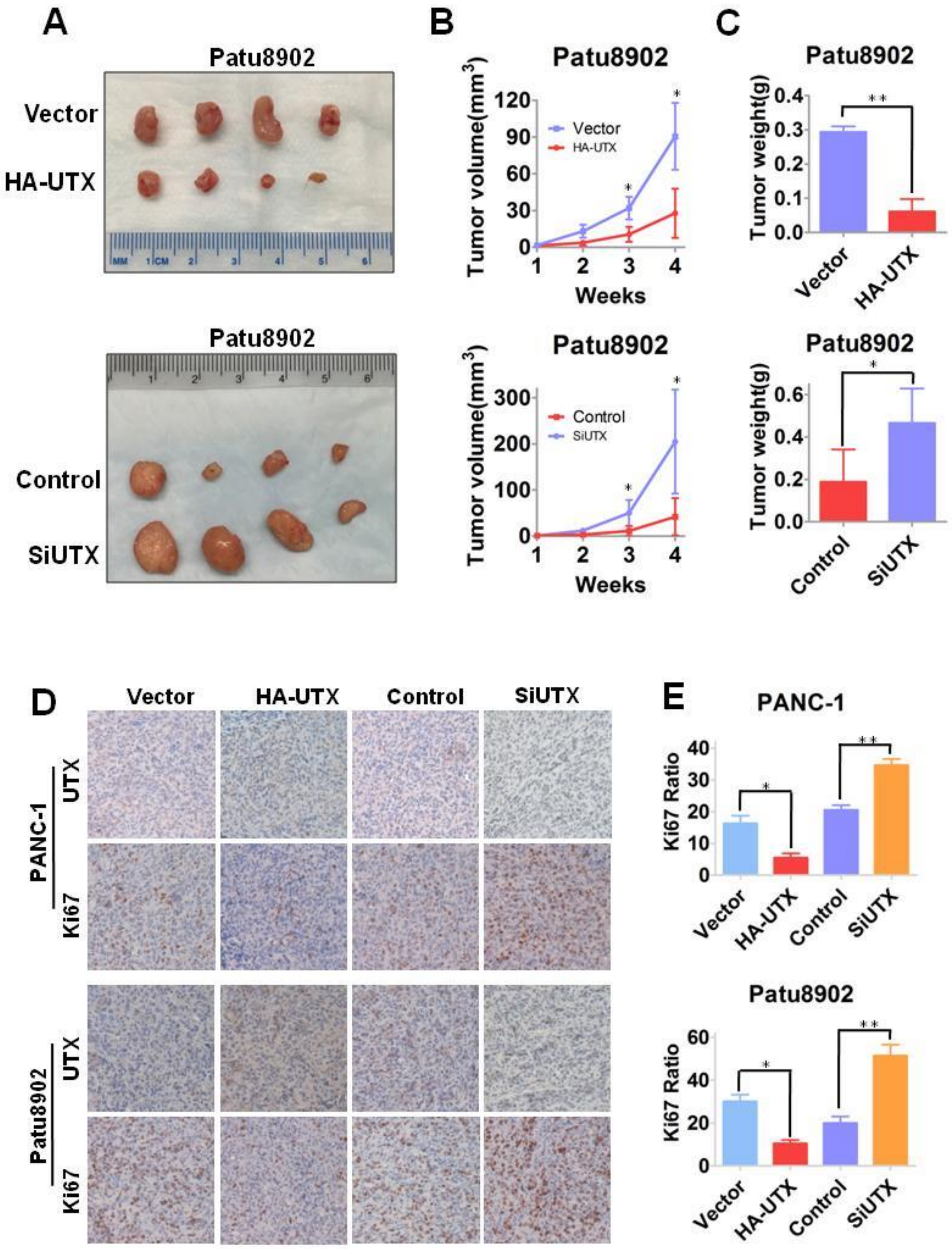

Figure S5. UTX inhibits tumor growth of PDA cells *in vivo*. A, Patu8902 cells with UTX overexpression or knockdown of UTX expression transfected with HA-UTX or siUTX were injected subcutaneously into the right scapular regions of nude mice (1×10^6 cells per mouse, four mice per group). Gross tumors in the mice (**A**), tumor growth curves (**B)**, and tumor weights (**B**) are shown. **D**, Stains of sections of tumors harvested from the mice described in Fig. 6 for specific antibodies against UTX and Ki67. **E**, Cell proliferation rates in the tumor sections in B. *$P < 0.05$; **$P < 0.01$.

Zhang *et al.*, Figure S6

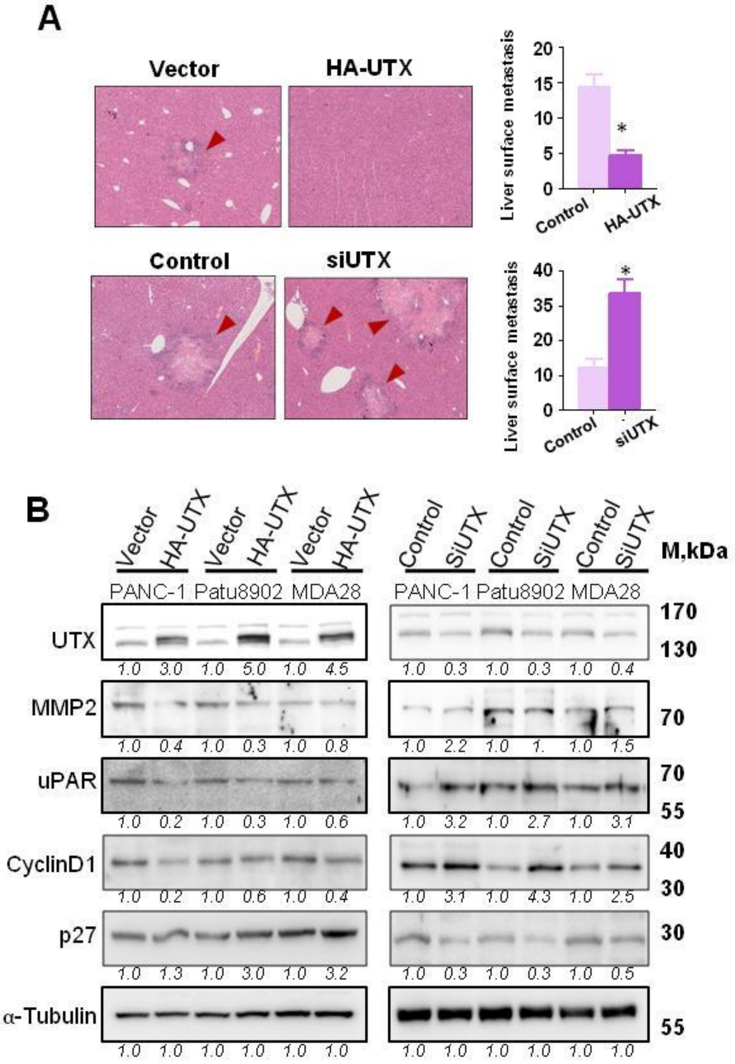

Figure S6. UTX inhibits tumor metastasis of PDA cells *in vivo*. A, PDA cells (PANC-1) with UTX overexpression or knockdown were injected intravenously into the ileocolic vein of nude mice (5×10^5 cells per mouse, five mice per group). Thirty-five days after injection, mice were killed, and livers were removed and processed for histological examination. Shown were hematoxylin- and eosin-stained sections of livers obtained from the mice (arrows indicated metastatic nodules); and the numbers of liver surface metastases were determined (right panels). **P*<0.05. **B**, PANC-1, Patu8902, and MDA28 cells were transfected with HA-UTX or siUTX and control vectors and siRNAs, respectively, for 48 hours. **A**, Western blot showing expression of UTX, MMP2, uPAR, cyclin D1, cyclin B1, p21, and p27 protein after upregulation or downregulation of expression of UTX in PDA cell lines.

A

JASPAR2018

Matrix ID	Name	Score	Relative score	Sequence ID	Start	End	Strand	Predicted sequence
MA1104.1	GATA6	12.3269	0.925768330268	UTX	1109	1121	+	tactgataagaca
MA1104.1	GATA6	8.67065	0.858453306165	UTX	970	982	-	ctgagagaagaca
MA1104.1	GATA6	8.20363	0.849854992677	UTX	372	384	+	cgtagaaaagatt
MA1104.1	GATA6	6.91287	0.82609100431	UTX	825	837	+	taaagattaaaag

B

AnimalTFDB3.0

Animal Transcription Factor DataBase Version 3.0

Results Filter by TF All

TF binding site Prediction Result

GATA6	hTFtarget	utx	1111	1119	+	13.6212	1.88e-05	0.0747	CTGATAAGA
GATA6	hTFtarget	utx	1111	1119	+	12.5429	5.26e-05	0.21	CTGATAAGA
GATA6	hTFtarget	utx	1111	1119	+	13.0909	2.88e-05	0.115	CTGATAAGA
GATA6	hTFtarget	utx	1110	1121	+	13.7576	6.52e-06	0.026	ACTGATAAGACA
GATA6	hTFtarget	utx	1111	1119	+	12.2571	2.77e-05	0.11	CTGATAAGA
GATA6	hTFtarget	utx	1110	1121	-	13	1.59e-05	0.0633	TGTCTTATCAGT
GATA6	database	utx	1107	1123	+	11.3537	7.96e-05	0.316	ACTACTGATAAGACACA
GATA6	hTFtarget	utx	1107	1122	-	13.4242	1.86e-05	0.0739	GTGTCTTATCAGTAGT
GATA6	hTFtarget	utx	1111	1122	+	12.5606	3.4e-05	0.135	CTGATAAGACAC
GATA6	hTFtarget	utx	1123	1134	+	13.6883	1.19e-05	0.0471	AGGGTAAACACA
GATA6	database	utx	1110	1119	+	8.82927	7.77e-05	0.309	ACTGATAAGA

Figure S7. Transcription factor prediction for UTX by using online analyzing tools. Putative binding sequences of GATA6 in *UTX* promoter were obtained from JASPAR (**A**) (http://jaspar.genereg.net/) and The Animal Transcription Factor DataBase (AnimalTFDB) (**B**) (http://bioinfo.life.hust.edu.cn/AnimalTFDB/#!/).

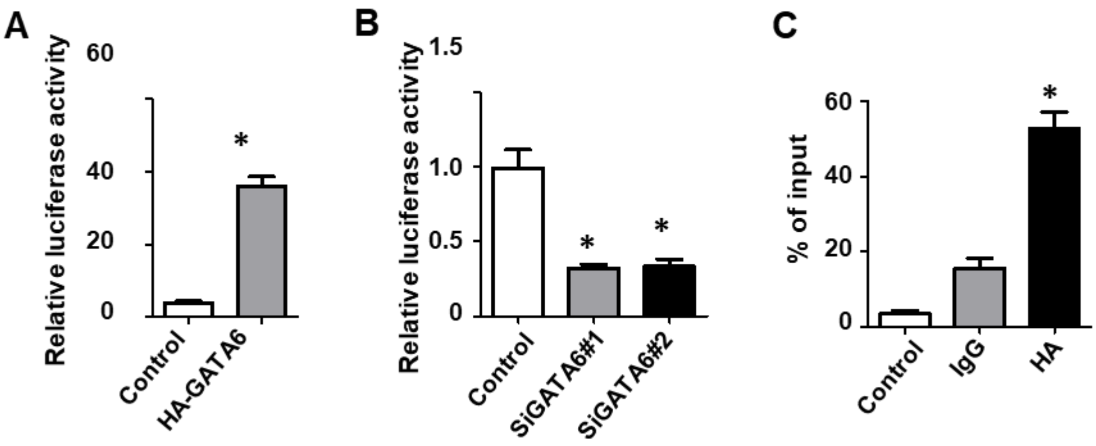

Figure S8. GATA6 directly activates UTX transcriptional activity. **A**, Patu8902 cells were co-transfected with p1799, GATA6 plasmid, or control vector. **B**, Patu8902 cells were co-transfected with p1799, siGATA6 (#1 or #2), or a non-targeting siRNA. The promoter activities in the cells determined using a dual luciferase assay are shown. **C**, Results of ChIP assay using chromatins isolated from Patu8902 cells. The immunoprecipitated DNA was analyzed by PCR followed by agarose gel electrophoresis. Genomic DNA input was 1%. The experiments were performed three times independently. *$P < 0.05$.
