# Supplementary material for: Loss of GATA6-mediated up-regulation of UTX promotes pancreatic tumorigenesis and progression
Source: Genes Dis. 2023 Mar 31;11(2):921–34. doi: 10.1016/j.gendis.2023.01.019 (PMC10491869; doi:10.1016/j.gendis.2023.01.019)
Supplement: Multimedia component 2 [file mmc2.pdf]

**Table S1.** Clinicopathologic characteristics of the 84 PDA patients

| **Characteristic** | ***n*** | **%** |
|---|---|---|
| Age | | |
| ≤60 years | 39 | 46 |
| >60 years | 45 | 54 |
| Sex | | |
| Female | 31 | 37 |
| Male | 53 | 63 |
| Differentiation | | |
| Well | 9 | 11 |
| Moderate | 42 | 50 |
| Poor | 33 | 39 |
| T Stage | | |
| T1 | 6 | 7 |
| T2 | 65 | 77 |
| T3 | 13 | 16 |
| N Stage | | |
| N0 | 53 | 63 |
| N1 | 31 | 37 |
| Metastasis | | |
| M0 | 82 | 98 |
| M1 | 2 | 2 |
| TNM Stage | | |
| I | 37 | 44 |
| II | 45 | 54 |
| III/IV | 2 | 2 |

**Table S2.** Clinicopathologic characteristics of the 84 PDA patients according to UTX expression levels

| **Parameter** | **All patients (*n*)** | **UTX expression (*n*)** | | ***P*** |
|---|---|---|---|---|
| | | **Low** | **High** | |
| Age | | | | 0.450 |
| ≤60 years | 39 | 24 | 15 | |
| >60 years | 45 | 26 | 19 | |
| Sex | | | | 0.493 |
| Female | 31 | 19 | 12 | |
| Male | 53 | 31 | 22 | |
| Differentiation | | | | 0.029 |
| Well | 9 | 3 | 6 | |
| Moderate | 42 | 22 | 20 | |
| Poor | 33 | 25 | 8 | |
| T Stage | | | | 0.071 |
| T1 | 6 | 2 | 4 | |
| T2 | 65 | 37 | 28 | |
| T3 | 13 | 11 | 2 | |
| N Stage | | | | 0.316 |
| N0 | 53 | 30 | 23 | |
| N1 | 31 | 20 | 11 | |
| Metastasis | | | | 0.351 |
| M0 | 82 | 48 | 34 | |
| M1 | 2 | 2 | 0 | |
| TNM category | | | | 0.242 |
| I | 37 | 19 | 18 | |
| II | 45 | 29 | 16 | |
| III/IV | 2 | 2 | 0 | |

The Pearson $\chi^2$ test was performed to calculate the statistical significance of the correlation between UTX expression level and patient characteristics.

**Table S3.** The sequences of the gene-specific primers used in this study

| **Gene name** | **Forward primer (5’-3’)** | **Reverse primer (5’-3’)** |
|---|---|---|
| Primers for deletion mutant promoter constructs of UTX | | |
| P1799(-1935~-1) | ACGAGGTCAGGAGTTGGAGAGCAG | ACGGCGGCGGCGTGGGGTTCGCTGT |
| P899(-1016~-1) | TGTTGGGTTCTATTGCCATGATTTC | ACGGCGGCGGCGTGGGGTTCGCTGT |
| P262(-383~-1) | TGGTGGGGAAGGGCGAGGTCCGGC | ACGGCGGCGGCGTGGGGTTCGCTGT |
| Primers for ChIP-PCR analyses | | |
| GBE#1 | ACGAGGTCAGGAGTTGGAGAGCAG | TCTATCTGTGCTTGTGCAAGTGTGT |
| GBE#2 | CATGATTTCAGTGGAGCCTTCTTAC | AGACAGACCTTTGGCGGTGGGGAGC |
